# Recovering Depth from Still Images for Underwater Dehazing Using Deep Learning

**DOI:** 10.3390/s20164580

**Published:** 2020-08-15

**Authors:** Javier Pérez, Mitch Bryson, Stefan B. Williams, Pedro J. Sanz

**Affiliations:** 1 Department of Computer Science and Engineering, Jaume I University, Vicent Sos Baynat, s/n, 12071 Castellón, Spain; sanzp@uji.es; 2 Australian Centre for Field Robotics, University of Sydney, Sydney, 2006 NSW, Australia; mitch.bryson@sydney.edu.au (M.B.); stefan.williams@sydney.edu.au (S.B.W.)

**Keywords:** deep learning, depth estimation, underwater, robotics, dehazing, image processing, 3D recovery

## Abstract

Estimating depth from a single image is a challenging problem, but it is also interesting due to the large amount of applications, such as underwater image dehazing. In this paper, a new perspective is provided; by taking advantage of the underwater haze that may provide a strong cue to the depth of the scene, a neural network can be used to estimate it. Using this approach the depthmap can be used in a dehazing method to enhance the image and recover original colors, offering a better input to image recognition algorithms and, thus, improving the robot performance during vision-based tasks such as object detection and characterization of the seafloor. Experiments are conducted on different datasets that cover a wide variety of textures and conditions, while using a dense stereo depthmap as ground truth for training, validation and testing. The results show that the neural network outperforms other alternatives, such as the dark channel prior methods and it is able to accurately estimate depth from a single image after a training stage with depth information.

## 1. Introduction

Underwater image dehazing is an interesting problem due to the increasing number of applications related to the underwater industry, for instance, the exploitation of offshore oil and deploying and maintaining underwater structures such as pipelines or underwater cables. Other possible use cases include maritime disasters, such as shipwrecks, leaks on offshore, or aircraft accidents.

Underwater intervention operations are usually performed by Remote Operated Vehicles (ROVs) controlled by expert pilots through an umbilical communication cable. However, in the last few years, a more autonomous architecture has been developed: Intervention Autonomous Underwater Vehicles (IAUV) [1]. These vehicles do not require a pilot and can deal with more reactive environments, such as object manipulation interventions, where faster movement corrections are required to grasp objects.

One of the main challenges that is faced by autonomous underwater vehicles is the need to interpret the highly unstructured and dynamic environment with which vehicle interacts. For instance, being able to detect and recognize the environment in degraded images to navigate or grasp and manipulate objects requires the ability to correctly understand the acquired image in real time with three-dimensional (3D) information to actually interact with it. Moreover the payload in an AUV is limited, restricting the hardware available.

In this paper, the estimation of depth from a single underwater image is addressed from the point of view of image dehazing for autonomous underwater vehicles. The approach uses a deep neural network to estimate a depthmap trained while using a dataset of images and ground truth 3D information acquired with a stereo pair. The main difference with previous work is the application and adaptation to dehaze underwater images, where haze may provide a strong cue to the depth of the scene. Because of its application in autonomous underwater vehicles, it is required to estimate depth-maps and dehaze images as fast as possible.

The paper is organized, as follows. Following Section 2 discusses the state of the art and the novelty of the approach. Section 3 describes the network architecture for depth estimation, the whole process for image dehazing and the experimental setup, including datasets used and evaluation metrics. In Section 4, the results obtained with the proposed solution are presented while in Section 5 a discussion of these results is presented and compared with state of the art methods. Finally, Section 6 concludes the work and proposes future work using the depth estimation in image dehazing.

## 2. State of the Art

### 2.1. Underwater Image Dehazing

Restoring degraded underwater images requires modelling and estimating many parameters, such as water absorption and scattering, which depend on a depth map. This work focusses on obtaining a fast depth map estimate in order to use it afterwards in a dehazing scheme. For a complete review on restoring images Raimondo et al. [2] and Han et al. [3] can be consulted.

Bryson et al. [4] use a whole dataset of images and depthmaps from the same survey in order to accurately estimate the parameters and restore the true colors of the image. Although this approach obtains a precise result, this technique requires a medium sized dataset of overlapping images and depthmaps of the same area that is not always available. Furthermore, it is not possible to use it in a real-time approach, as it is computationally expensive and requires the whole dataset to dehaze any image of it.

Recent work has examined the use of Markov Random Fields (MRF) and a training stage to learn how to assign the most probable color to each pixel, such as Torres-Méndez et al. [5]. However, this method ignores the effect of the depth in the attenuation process, learning fixed transformations for objects independently of its depth making it imprecise for object detection which is one of the goals of autonomous underwater vehicles.

Roser et al. [6] propose a method using a stereo camera in order to obtain a depthmap and use this depthmap and a single image to estimate the rest of parameters to dehaze the image. Once again, the method depends on a dense depthmap that may not be available when the environment is not textured enough for the stereo camera to recover 3D information, such as mud or sand, commonly present in underwater images. Although the work is interesting, it is not feasible when only a monocular camera is available. Moreover, due to the complex computational minimizations computation time being too high to include it in autonomous underwater vehicles, even in small images (640 × 480), a single image enhancement requires 2.5 s.

Other alternatives use specific hardware, such as Vasilescu et al. [7], which dynamically mix the illumination of an object in a distance-dependent way by using a controllable multicolor light source to compensate color loss. The main problem of this kind of solutions is the need of a specific hardware to solve the problem that increases vehicle payload, hardware complexity, and costs.

Finally, many works use a single image to estimate these parameters based on the Dark Channel Prior (DCP) [8]. Dark Prior is based on the observation that, in outdoor haze-free images in most of the non-background patches, at least one color channel has some pixels whose intensity is very low and close to zero. This works in most outdoor air images and has also been used in underwater environments such as the Bianco Prior [9], underwater dark prior citeunderwaterDarkPrior, wavelength compensation and dehazing [10], and the modification by Drews et al. [11]. The main disadvantage of this method is that it is based on a statistical observation that may not be valid for some cases and many works rely on subjective visual results instead of objective numerical validation. However, it has the requirements for the image enhancement to autonomous manipulation case proposed: single image input, image restoration, and real time performance.

The european project iMARECULTURE [12], which is aimed to “bring inherently unreachable underwater cultural heritage within digital reach of the wide public using virtual visits and immersive technologies”, also provides an study of different underwater image enhacement methods in Mangeruga et al. [13]. This study compares different enhancement methods, concluding that ACE [14] and CLAHE [15] algorithms lead to the best results in most situations. These two methods have been used in the qualitative results to compare with the proposed dehazing application.

Because of the great impact of deep learning in the last few years, some authors proposed different machine learning approaches for image dehazing. However, the impossibility of obtaining good pairs of hazy and haze free images usually force the authors to use synthetically generated images, such as Hussain et al. [16], Cai et al. [17], Tang et al. [18], Zhu et al. [19], or [20]. These works use different machine learning approaches, such as deep MLP networks, CNN’s, random forests, or supervised learning. However, these synthetic images do not include all the complexity of real underwater images, and approaches based on them may not obtain good results in real environments.

### 2.2. Depth Estimation

Previously cited works struggle finding a suitable transmission estimation for image dehazing. Authors mention the possibility of using a depth-map as transmission is linearly related to it, reducing the problem complexity estimating a single wave-length dependent parameter instead of a transmission estimation for each pixel. Furthermore, the 3D environment helps in recognition tasks and, as a consequence, in subsequent stages. This is the reason why there is much prior work on sensors that are able to sense this kind of information, such as stereo cameras, motion cameras, and infrared camera/projector (popularly known as Kinect). For these reasons, being able to estimate 3D from still images is a key value beyond the single image dehazing capability.

Unfortunately obtaining a good depth-map is not always possible to acquire with sensors, and this is especially true in underwater environments; infrared projectors do not work underwater and stereo or motion cameras may not be able to find enough features to recover 3D.

Furthermore, the depthmap information may still be impossible to retrieve in many situations, for example, where the seafloor is not textured enough to obtain features that can be recovered and, even when the system is capable of doing this, the depthmap will still have gaps or zones without depth information. However, under controlled situations, it is possible to acquire a ground truth dataset to be used in a machine learning scheme.

One of the first works to address this problem from a learning perpective was Saxena et al. (2008) [21], extended in Saxena et al. (2009) [22], which uses a linear regression and a MRF for predicting the depth in a set of images. However, the system relies on horizontal alignment of images that cannot be assured in the proposed application.

In Eigen et al. (2014) [23], a deep convolutional network is used to estimate 3D depth in different images. This approach takes a single RGB image and produces a depthmap using two networks. The first one denominated “coarse”, predicts a global depth with five convolutional and pooling layers, followed by two fully connected layers reducing the size of the image. The result of this network is concatenated in a “fine” network with the input image in three additional convolutional and pooling layers to obtain an output of 1/4 resolution. The loss function is a scale invariant mean squared error.

The previous work was further extended by Eigen et al. (2015) [24] also using the concept of coarse and fine estimation adding a new scale network for even higher resolution obtaining output of half the input resolution. This work adds gradients to the scale invariant mean squared error loss function. Besides depth, it also uses the network in order to predict normals and per-pixel semantic labeling.

Ladicky et al. [25] also simultaneously performs depth estimation and semantic labelling on the same dataset. It can be concluded that doing it at the same time benefits both processes improving the results, knowing that the label helps to estimate the depth and vice versa. However, this method uses hand crafted features that may not be useful in other contexts.

Finally, Baig et al. [26] use a slightly different approach for refining the results while maintaining a convolutional deep network for coarse estimation. The local refinement directly regresses on pixel depth while using the global estimate as a feature.

## 3. Material and Methods

In this paper, different neural network architectures are tested to finally propose a similar approach to Eigen et al. (2015) [24] substituting the network in the refine step for a guided filter [27] that produced better performance. In order to evaluate the results, different metrics are used, demonstrating that the obtained depthmap is suitable for image dehazing.

The proposed method consists of three steps: (1) obtain a coarse estimation of the depthmap using a neural network, (2) refine the estimation with a guided filter, and (3) the final application: dehaze the image using this estimation by combining this estimate with an inverse underwater image formation model based on attenuation.

### 3.1. Coarse Estimation of the Depth Map Using a CNN

The first step, coarse estimation, is performed through a convolutional neural network. Once trained, the neural network receives an RGB image as input and produces a rough depthmap. The training stage also requires depth estimation, so the network is able to learn depth cues from the images.

Even though monocular images do not contain direct data on the absolute depth to objects in the scene, within a constrained set of environmental parameters various visual cues may allude to relative scene depth, such as shading and variations in contrast, caused by light moving through different distances in the water column. A key idea of this paper is that such cues may be learned for a particular environment using a deep learning framework. The main problem is that the task is inherently ambiguous; given an image, an infinite number of possible scenes may have produced it, making impossible to decide the scale. This fact refers to the impossibility to distinguish a real house from a dollhouse in a two-dimensional (2D) picture. Although relative object sizes can be inferred using visual cues, such as shadows, lines, light attenuation, etcetera, concluding that an object is further than other one, the absolute size of the objects is still uncertain.

To address this, scale-invariant metric is used in the training stage as a loss function, as explained in detail in the following section. This function has a coefficient to balance between obtaining an absolute value and correctly estimating the depth structure.

The goal of this network is to predict a depth map using the whole image. In order to do so, the image is processed through multiple convolutional and pooling steps. In this case, fully connected layers were not used in order to maintain the image size as big as possible.

**Figure 1:**
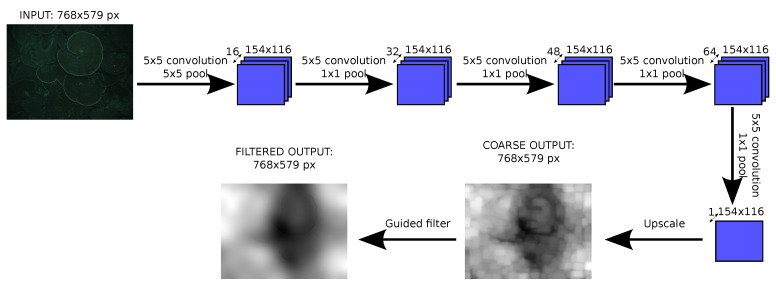
Architecture of the convolutional network used to estimate depth.

As Figure 1 shows, five layers of convolution and pooling have been used. The first layer reduces the image size with a 5 × 5 pooling, while the others maintain the size. The number of filters increase in each layer until the last one that is reduced to one, the output.

After the last convolutional layer, a bilinear upscale is performed to recover the original size, and this result is sent to the guided filter. The resulting upscaled image is used to train the neural network, such that the network learns the effect of the upscaling.

All of the hidden layers use rectified linear unit (ReLu) [28] activation, with the exception of the last convolutional layer, which is linear. The ReLu activation function is defined as f(x)=max(0,x) and shows a faster learning rate than sigmoids and hyperbolic tangents functions. The Adam optimizer [29] has been used in order to train the neural network.

Regarding the training procedure, minibatches of 10 images were used and an early stopping was used to decide the optimal moment to stop training. The early stopping monitored the loss value for a small validation subset from the training set. In the case that this value increases or stabilises after a few epochs, the training is stopped and the best neural network for the small validation subset is used.

### 3.2. Loss Function

The loss function used to train the neural network of the step (1) is a scale invariant error, as proposed in other works for depth estimation in single images. Accordingly, the training loss per image is set to Equation (1).

(1) $$L\left(gt,y\right)=\frac{1}{n}\sum_{i}d_{i}^{2}-\frac{\delta }{n^{2}}{\left(\sum_{i}d_{i}\right)}^{2}$$

where $d_{i}$ is the log difference between estimation $y_{i}$ and groundtruth $gt_{i}$: $d_{i}=\log y_{i}-\log gt_{i}$, *n* is the number of pixels and δ∈[0,1]. The first part of the equation is the metric learning part computed as the mean squared logarithmic difference, while the second part is a structure learning term that benefits if the errors have the same sign and penalises otherwise. This means tha single depth misestimations are not penalised if the rest of the pixels are misestimated in the same proportion. The δ term is a coefficient that controls the amount of structure term. In this work, 0.5 showed a good balance between minimizing the error and learning structure.

Additionally, adding gradients errors, first derivative, has been tested as proposed in Eigen et al. (2015) [24]. This forces the resulting depth-map to match not only absolute values, but also depth slopes in the X and Y axis, although adding a significant amount computational complexity.

Groundtruth depthmaps were acquired while using stereo cameras. For this reason, some points in the depthmaps do not have a valid depth. In order to deal with this, the loss function is only evaluated in points with valid depth, adjusting *n* for each image, and performing the sums only when depth is available.

### 3.3. Estimation Refinement through Guided Filtering

Once the coarse estimation is done through the neural network, a blurred image containing the estimated depthmap can be obtained. It is necessary to refine this result in order to keep details of the original image. Different strategies have been tested, such as the fine-scale network used in Eigen et al. (2015) [24], a gaussian filter, or modifying the network to work with full-scale images. However, the one that produced the best results, by far, was using a guided filter [27].

**Figure 2:**
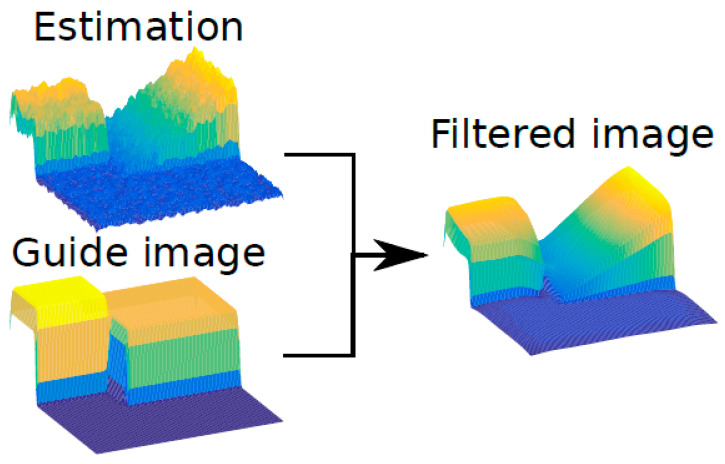
Guided filter example.

The guided filter is an image process where the output is computed when considering the content of a guidance image, see Figure 2. This is supposed to filter the image smoothing it but preserving the edges present in the guide image. This effect can be seen in Figure 2, the estimation is smoothed respecting the edges in the guide image. As can be seen the random noise in the estimation surface has almost disappeared in the filtered image, but it still preserves the edges that are already present in the guide image.

In this case, the input of the guided filter is the depthmap estimation calculated by the neural network and the guide used is the original image. This step is similar to the one used in dark channel prior (DCP) [8] with an image matting. The main idea of using it like this is to soften the noise on the estimation while preserving the details that are already present in the image.

### 3.4. Image Dehazing

The final step uses the estimated refined depthmap as input in another process; in this work, an image dehazing application is proposed. A simple solution analogue to DCP [8] has been chosen to make it possible to work in real time, although more advanced techniques may achieve better results. This simple dehazing mechanism has been chosen to show an application of the depthmap, but the main contribution of this work is obtaining an accurate depthmap from single images.

As a depth-map estimation *d* is available, it is used to estimate transmission $\tilde{t}$, multiplying it by a constant attenuation b(λ). This procedure avoids the DCP transmission estimation which is based in the observation that in most of the non-background patches, at least one color channel has some pixels whose intensity is very low and close to zero. This observation may not be true in some underwater images, and depth-map is much more solid evidence for transmission estimation.

The value of the most distant pixel (RGB) proved to be a good estimator for computing attenuation b(λ) as proposed by the DCP algorithm. This value is computed in each channel as the light attenuates, depending on wavelength in underwater environments. In order to compute b(λ), DCP assumes the haziest pixel to be equal to the atmospheric light *A*, and assuming a uniform distribution of the scattered light γ(λ), a rough estimation of attenuation can be obtained through Equation (2), where *L* is a the constant light that gets attenuated.

(2) $$A=\frac{\gamma (\lambda )L}{b(\lambda )}$$

Using this, the image can be processed to recover the original colors from the attenuation. Accordingly, the inverse of the simplified attenuation function (3) is used, being *I* the image acquired by the camera, *J* the image without noise.

(3) $$I=J\tilde{t}\left(x\right)=Je^{-b\left(\lambda \right)d}$$

Just using the previously estimated values for depth *d* and attenuation b(λ) and the captured image *I*, it is possible to restore the “original” image *J*. Finally, a histogram equalization is applied in order to enhance the colors of the image.

### 3.5. Materials: Image Datasets

In this work, different datasets have been used to train validate and finally test different methods for image restoration. Each dataset consists of a group of images taken at the same survey. Along with each image, a dense stereo depthmap has also been gathered providing information for almost every pixel about its depth. The information associated with each image allows for the approaches to be benchmarked and provides training data for the image restoration methods.

Five different datasets have been used to cover a good variety of backgrounds, illuminations, and turbidity conditions. However, all of the image datasets share a common trait: cameras are pointing to the seafloor, as they were acquired from an autonomous underwater vehicle, which is the final application system of this work.

**Figure 3:**
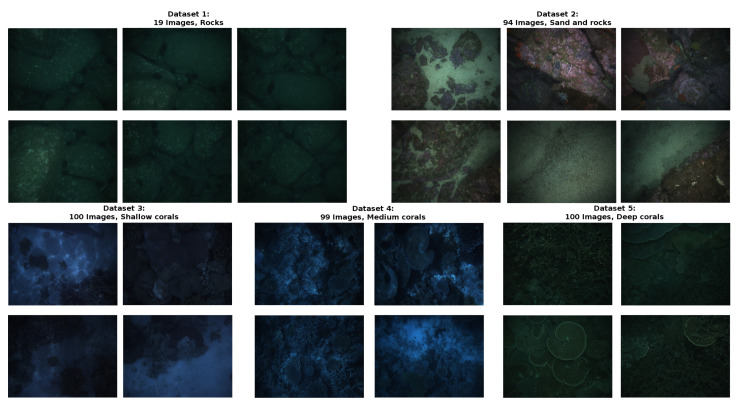
Images of the different datasets used in the work.

Figure 3 shows example images from each dataset. Images cover a different range of textures, vehicle depths, illumination conditions, and distance from the camera to the seafloor. Table 1 shows the specific details of each dataset.

The images were acquired by the AUV Sirius [30] at five different field locations across Australia. Datasets 1 and 2 were acquired over boulderfields at St Helens, Tasmania. Datasets 3 to 5 were acquired over coral reefs at One Tree Island (dataset 3) and Heron Island (dataset 4), on the southern Great Barrier Reef and Houtman Abrolhos Islands, Western Australia (dataset 5). All of the images were captured while using a calibrated stereo-pair consisting of two prosilica 1.3 MPix cameras. During datasets 1, 2, and 5, artificial lighting was provided by two xenon strobes mounted to the AUV (datasets 3 and 4 were captured in shallow waters and illuminated by sunlight). All of the stereo pairs were post-processed and combined with adjacent images pairs to produce a feature-based stereo depth map as described in Kingma et al. [31], reprojected back into each camera, with a spatial resolution of 2.5 cm, and sub-centimeter depth accuracy, based on analysis of residual feature errors.

While training, these datasets have been augmented with random transformations, so it can generalize a model and abstract local details, like specific image positions of depth cues. Images are randomly flipped horizontally and vertically with 0.5 probability.

The five original datasets have been divided in different subsets that will be used for training, validating, and finally testing in order to maximize the data usefulness. To evaluate the performance of all the datasets equally, 20% of the images have been left from the training set of each dataset.

Following the best practices, training and validating different network architectures and hyperparameter tuning has been conducted exclusively on dataset 5, deep corals. The validation of these experiments was carried out using only the images left apart from this dataset.

The final results were obtained using the resting original datasets (1–4) as test, while training included images from all the datasets.

### 3.6. Metrics and Evaluation

Because of the nature of the application, three different metrics are used to check the validity of the estimation: Correlation, Root Minimum Squared Error (RMSE), and Adjusted Root Minimum Squared Error (ARMSE). To measure correlation the sample Pearson correlation coefficient Equation (4) for a sample is used, where *x* and *y* are estimated depth values and true depth values respectively. It measures the linear correlation between two variables X,Y giving a value between −1 and +1 where 1 is total positive correlation, 0 is no correlation and −1 is total negative correlation.

(4) $$r_{xy}=\frac{\sum_{i=1}^{n}\left(x_{i}-\bar{x}\right)\left(y_{i}-\bar{y}\right)}{\sqrt{\sum_{i=1}^{n}{\left(x_{i}-\bar{x}\right)}^{2}}\sqrt{\sum_{i=1}^{n}{\left(y_{i}-\bar{y}\right)}^{2}}}$$

This metric is useful to know whether both surfaces are similar in terms of appearance. As neural networks are trained to minimize the error between surfaces the result should be close to 1, the higher the better. This metric is not dependent on scale, so it is not sufficient to conclude whether the proposed solution is accurate.

The second proposed metric is a Root Minimum Squared Error. It is actually one of the terms of the minimization function, so the lower the better. In this case, it is measuring the point-to-point error, so surfaces could be different in shape but still have a good RMSE value.

Finally, an Adjusted Root Minimum Squared Error is proposed. This measure equals the means of the estimation and ground truth so the scale uncertainty problem, is avoided given that both depthmaps now have the same scale. This is a good measure for the dehazing problem, as the depthmap is multiplied by the attenuation, which has to be estimated. So the scale of the estimation is not so important as in other applications.

In addition, these last two metrics are also computed relative to the ground truth depth, so a percent error is obtained. The equations to calculate this error for an estimation *x* and groundtruth *y* measures can be seen in Equation (5), using an adjusted estimation x=Ax computed like $Ax_{i}=\frac{x_{i}\bar{y}}{\bar{x}}$ provides ARMSE.

(5) $$\begin{array}{c} RMSE=\sqrt{\frac{1}{n}\sum_{i=1}^{n}{\left(x_{i}-y_{i}\right)}^{2}}\\ RRMSE=\frac{1}{n}\sum_{i=1}^{n}\left|\frac{\left(x_{i}-y_{i}\right)}{y_{i}}\right| \end{array}$$

**Figure 4:**
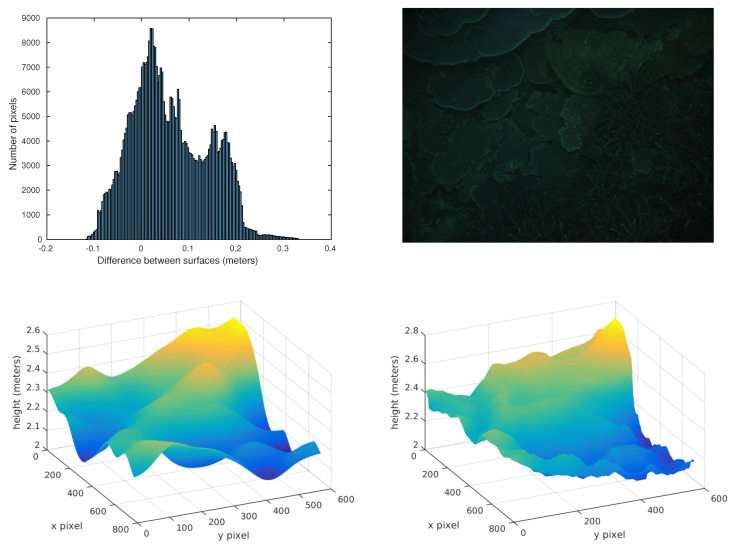
Visual metrics and evaluation: (**top right**) Visualization of the image, (**top left**) error histogram, (**bottom right**) “true” depthmap surface and (**bottom left**) neural network estimation surface.

In terms of visualization, 3D plots of the estimated and groundtruth surfaces, pixel error histograms, and RMSE can be obtained similar to the one that is shown in Figure 4. For instance, in this figure, the top left bar chart shows a histogram of the depth estimation errors for the top right image. The bottom surfaces are the stereo ground truth depth map on the right side, and the neural network estimation on the left side. This kind of visualization has been used to check the validity of the metrics and the performance of the neural network.

In order to compare with other state-of-the-art algorithms, besides other neural network approaches, the DCP technique [8] has been used in the image datasets. The correlation and ARMSE metrics are also computed and shown. In order to compute the ARMSE metric it is necessary to use the transmission estimation of the DCP shown in Equation (6), where b(λ) is the attenuation and *d* the depthmap. The optimal b(λ) has been used to obtain the depthmap to compare with the groundtruth.

(6) $$\tilde{t}\left(x\right)=e^{-b\left(\lambda \right)d}$$

As discussed in the state of the art, other methods have been discarded either because they are not suitable for real time application in autonomous underwater vehicles or require additional hardware that increases the payload and complexity and do not have an available dataset to compare with.

Finally, the dehazing step only shows qualitative results of known techniques, such as DCP, histogram equalization, CLAHE [15], and ACE [14], due to the impossibility of obtaining groundtruth pairs of raw-dehazed images in order to obtain suitable metrics.

## 4. Results

To show the validity of the depth estimation, three experiments have been conducted. The first is the comparison of different neural network architectures and the dark prior estimating the depth of images using only dataset 5. During this experiment neural network hyperparameters are tuned. The second experiment extends the images used to the five datasets, in order to validate the results that were obtained in dataset 5. The last experiment uses the estimation in the potential application of single image dehazing.

### 4.1. Single Dataset Depth Estimation

The results using only dataset 5 are shown in Table 2 for different network architectures. The first results correspond to the proposed architecture, *noguided* is the same neural network, but skipping the guided filter. *coarsefine* is using the structure presented in Eigen et al. (2014) [23]; the next result is the same network plus a guided filter. Different choices for the number of convolutional layers are shown. Adding gradient error to the loss function is also tested, as proposed in Eigen et al. (2015) [24]. Finally, *nostrides* is the architecture that does not reduce the image size while convoluting. Additionally, the dark channel prior results are shown in order to compare with a non-learning approach.

The first noticeable thing is that using a guided filter helps to improve the results. Even if the network has a part devoted to it, as in the case of the coarsefine network, using the guided filter enhances the results. In terms of the number of layers, five layers appears to be the optimum value, four layers does not allow the system to capture the complexity of the problem, and more than five starts to overfit, reducing the train error but increasing the validation error.

Using the gradients in the loss function shows no benefit, the results are very similar to the ones achieved by the neural network without them. The main drawback is computing gradient errors increased the training time around 50%, even with precalculated gradients for ground truth images, increasing memory consumption in 400% (gradients and valid gradients in X and Y axis).

The nostrides solution works with the complete image through all layers, as a consequence training time is much higher. However, this longer training time does not pay off in terms of results. Reducing the image size with pooling and scaling the estimation back produces better results.

Dark channel prior results are added in order to compare with a state-of-the-art dehazing method suitable for single image dehazing. As the transmission estimation method is not based on a depth-map, only adjusted results can be used to evaluate, while assuming the best possible attenuation to compute the depth-map. Even in this situation the results are far from the obtained using neural networks.

The best results achieved show a 0.7909 correlation which is a strong correlation, around 10 cm mean error without scale adjust and 8 cm adjusting it. The relative error is between 4% and 3.1%, depending on the scale adjustment.

### 4.2. Multiple Dataset Depth Estimation

Taking into account the previous results, the neural network has been trained using all of the datasets in a second experiment, only for validating the most promising architectures. The results obtained can be seen in Table 3. The proposed architecture results are showed first, followed by the same network without a filtering step. The next results correspond to the coarse and fine network without and with guided filter. The last neural network compared is the non-reducing architecture and, finally, dark prior results are added to the comparison.

As can be seen, the proposed architecture offers the best results when all of the datasets are mixed. In comparison with the previous results corresponding to only one dataset, the RMSE error increases significantly while the adjusted results are similar.

The results show that the guided filter is a good choice for enhancing the results. It reduces the noise while keeping the surface edges using the original image. The network that does not reduce the image obtains the worst results from the different architectures tested and the one presented in Eigen et al. (2014) [23] is significantly worse than the one presented here.

**Figure 5:**
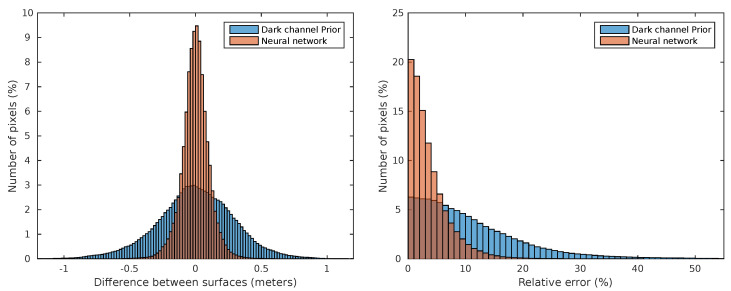
Error histograms, ARMSE (**left**) RARMSE (**right**), for all the images pixels comparing neural network and dark channel prior.

Finally, the dark channel prior estimation results are also far from the ones that are achieved by neural networks. This is especially true in correlation metric, where dark prior shows no correlation with depthmap while the learning solution shows a strong relationship. In Figure 5, adjusted errors and relative errors for each pixel in each image are depicted in a histogram chart comparing DCP and neural network. The left histogram represents the percent of pixels of every validation image in the y axis and the depth estimation error in the x axis. The right histogram corresponds to the relative error instead of absolute error. As can be seen, the neural network obtains much better results, showing a smaller deviation in results.

### 4.3. Qualitative Results

**Figure 6:**
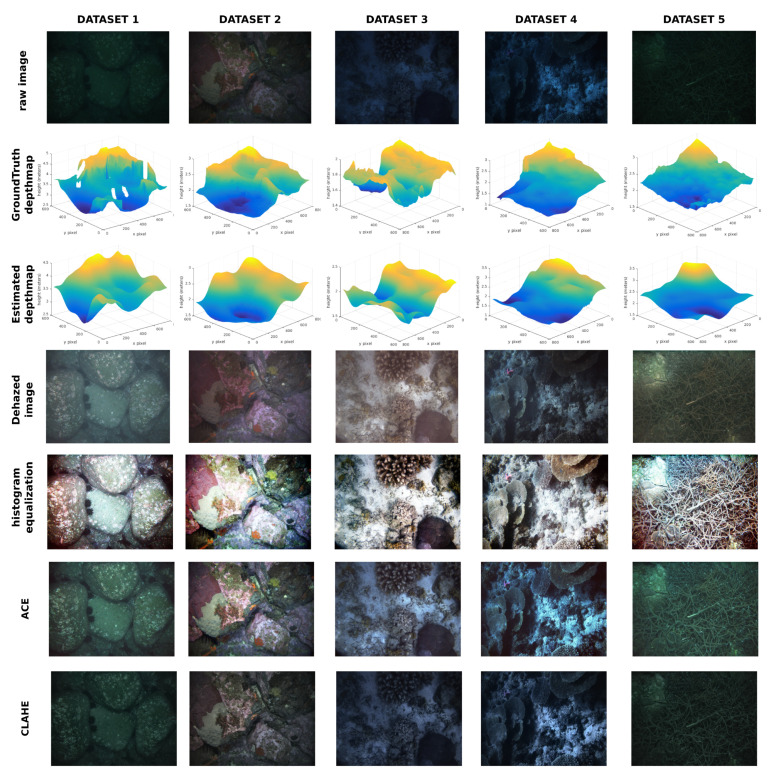
Neural network results applied to image dehazing, from left to right dataset images, from top to bottom raw image, groundTruth depthmap, neural network estimated depthmap, dehazed image using the estimated depthmap, histogram equalized image, Automatic Color Enhancement (ACE), and Contrast Limited Adaptative Histogram Equalization (CLAHE).

Due to the impossibility of acquiring a complete hazy/dehazed real images dataset, qualitative results are shown in Figure 6. In this figure, one representative image and groundtruth depthmap of each dataset can be seen with the results of depthmap estimation, image dehazed, histogram equalization, and two more specific state of the art restoration processes, such as Automatic Color Enhancement (ACE) [14], and Contrast Limited Adaptative Histogram Equalization (CLAHE) [15].

DCP has been omitted from qualitative results, because it produced faulty results in the tested images, as it was not designed for underwater dehazing, as shown in a previous depth estimation evaluation.

The estimated surfaces are similar to the ground truth provided, lacking small details, but filling the gaps where stereo was not able to recover 3D.

## 5. Discussion

The main advantage of the presented methodology for depth estimation is that it is possible to use it in stereo cameras to fill the gaps when there is no texture as well as in monocular cameras. Furthermore, the proposed approach and the DCP based solutions are the only capable of working in a real time single image system without additional hardware. Real time performance is a basic requirement for its use in autonomous underwater vehicles, where navigation and performance completely depend on a correct interpretation of images in real time. Using additional hardware increases the vehicle payload and makes the system more complex. For these reasons, the proposed method is directly compared with DCP solutions, although the results from other works will be mentioned.

Although matching not only depths but gradients, slopes from a 3D perspective, seems an interesting feature, the use of image gradients in the loss function showed no benefit. Moreover, calculating image gradients is highly time and memory consuming. Taking into account real time performance is desirable, this solution was discarded, but it is still a feasible area for future work.

The proposed depth estimation algorithm is capable of estimating depth with a 5.3% with respect to groundtruth in absolute terms and 3.7% adjusting the scale. Scale adjusted error is useful as many underwater vehicles use sonar beams to obtain single depth measures that would allow for adjusting depth-map scale in a real situation.

The results from multiple datasets are slightly worse for non-adjusted metrics than the achieved for just a single dataset, supporting the inherent scale problem stated before. When images are just from one location, the conditions through all the images are similar and scale is easier to estimate. In any case, the achieved results are good enough for most applications, such as image dehazing, in absolute, and scale invariant cases.

It is also interesting to mention the results of Eigen et al. (2014) [23] in open air images, which achieved around 21% of RRMSE and 0.9 meters of RMSE. When comparing this to the ones achieved on underwater images, it shows that the depth cues in single underwater images are much stronger than in open air. This makes it possible to retrieve 3D information and then use it to understand the scene and finally improve autonomous vehicles performance.

**Figure 7:**
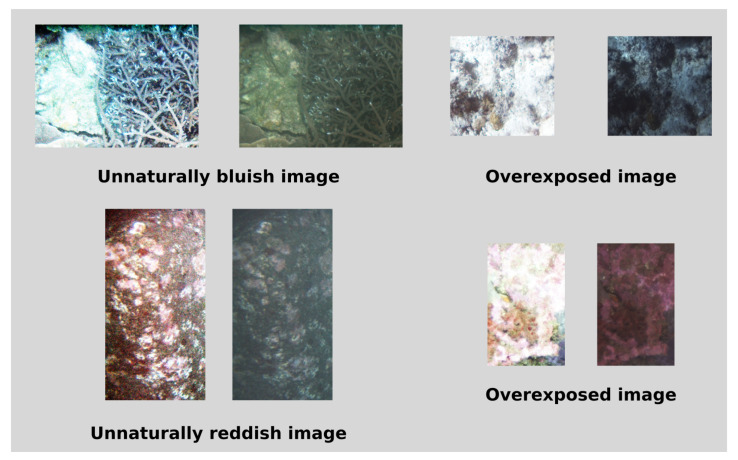
Comparison of histogram equalized only (**left**) and previously dehazed image (**right**) in images that produced different artifacts.

Regarding image dehazing, differences between proposed method results and the different compared alternatives can be seen. Proposed approach dehazed images look more natural and perform better when there are big differences in depth, as can be seen in the dataset 2 image, where the histogram equalized image is brighter in parts closer to the camera and darker in farther depths. The histogram equalization on the last image also fails to restore original colors, obtaining non-natural colors due to overamplification of noise. This effects can be seen in Figure 7.

In the case of ACE, the algorithm is not able to completely remove the original colors of the water, images in the first and fifth dataset look greenish, while third and fourth look bluish. With the CLAHE dehazing, the results are similar, but the images look darker than the ACE algorithm. On the other hand, the proposed solution images look more natural, only dataset 5 keeps greenish

## 6. Conclusions

A new 3D depth estimation from single underwater images methodology has been presented. The performance of this method is discussed and compared in the computation of image dehazing for single image oriented to enhancing autonomous missions, although other applications are feasible.

The main advantages of this technique are the requirement of only a single image as input, which produces a depthmap with the same resolution as input and does not require texture features as stereo.

The obtained results show depth cues in underwater image provide more information than in open air images. Underwater neural network achieves 5% error in underwater images, while a similar network described in Eigen et al. (2014) [23] obtained 21% in open air images. This is probably caused by light attenuation and underwater haze.

More advanced techniques may be applied to the dehazing scheme that exploit the image and estimated depth-map from the neural network so the dehazing is better; however, this is left as future work. Other possible extensions to this work include feeding the neural network with seafloor depth or sunlight information, this will probably make the system more precise at depth estimation.

## Figures and Tables

**Table 1:** Description of datasets.

Description	Images	Seafloor Depth	Image Range	Mean Distance	Strobes
Rocks	19	27 m	2.18–4.78 m	3.64 m	Yes
Rocks sand	94	27 m	0.46–4.17 m	2.29 m	Yes
Shallow corals	100	2 m	0.34–2.73 m	1.91 m	No
Medium corals	99	7 m	1.06–4.77 m	1.50 m	No
Deep corals	100	18 m	0.97–4.83 m	4.03 m	Yes

**Table 2:** Results of different neural network architectures on dataset 5.

Network	Correlation	RMSE (m)	RRMSE (%)	ARMSE (m)	RARMSE (%)
Proposed	**0.7909**	**0.1003**	3.9471	**0.0819**	**3.1481**
Noguided	0.7506	0.1099	4.2939	0.0933	3.5524
coarsefine [23]	0.7101	0.1196	4.6446	0.1015	3.8186
coarsefine guided	0.7585	0.1074	4.2184	0.0877	3.3598
7 layers	0.7597	0.1089	4.1839	0.0923	3.5171
6 layers	0.7692	0.1101	4.3343	0.0894	3.4692
4 layers	0.6705	0.1399	5.8538	0.1044	4.0513
gradients [24]	0.7888	0.1015	**3.9469**	0.0834	3.2055
nostrides	0.7448	0.1207	5.0060	0.0878	3.3865
Dark prior [8]	0.0464	-	-	0.1893	7.4065

**Table 3:** Results of different neural network architectures using all the datasets.

Network	Correlation	RMSE (m)	RRMSE (%)	ARMSE (m)	RARMSE (%)
Proposed	**0.8181**	**0.1293**	**5.2632**	**0.0982**	**3.6668**
Noguided	0.7878	0.1430	5.6955	0.1141	4.2351
coarsefine [23]	0.7047	0.1653	6.0099	0.1342	4.5998
coarsefine guided	0.7447	0.1508	5.5593	0.1169	3.9949
nostrides	0.6904	0.1816	7.3358	0.1214	4.6501
Dark prior [8]	0.0737	-	-	0.2788	10.8783

## Abbreviations

The following abbreviations are used in this manuscript:

ROV	Remote Operated Vehicle
IAUV	Intervention Autonomous Underwater Vehicle
ReLu	Rectified Linear Unit
DCP	Dark Channel Prior
RMSE	Root Mean Squared Error
ARMSE	Adjusted Root Mean Squared Error
RRMSE	Relative Root Mean Squared Error
RARMSE	Relative Adjusted Root Mean Squared Error
